# Functional status and caregiver burden of patients on maintenance haemodialysis in Cameroon: a two-centre cross-sectional study

**DOI:** 10.1186/s12882-022-02977-3

**Published:** 2022-10-22

**Authors:** Alex Tatang Mambap, Innocent Abong Che, Maimouna Mahamat, Gloria Enow Ashuntantang

**Affiliations:** 1 Department of Clinical Sciences, Faculty of Health Sciences, The University of Bamenda, Bamenda, Cameroon; 2 Bamenda Regional Hospital, Bamenda, Cameroon; 3 Department of Internal Medicine And Specialties of the Faculty of Medicine and Biomedical Sciences, The University of Yaounde I, Yaounde, Cameroon; 4 Yaounde General Hospital, Yaounde, Cameroon

**Keywords:** Functional status, Functional status impairment, Caregiver burden, Maintenance haemodialysis

## Abstract

**Background:**

Data on the functional status (FS) of patients on maintenance haemodialysis (MHD) and their caregiver burden (CGB) in SSA where patients have fewer weekly dialysis sessions and pay out-of-pocket for dialysis-related costs is sparse.

**Objectives:**

To assess the functional status of Patients on MHD in Cameroon, and the burden of their Caregivers, and to determine the factors associated with functional status impairment (FSI), and high caregiver burden (HCGB).

**Methods:**

We consecutively enrolled patients on MHD at the Bamenda Regional, and Yaounde General Hospitals over a period of 3 months. We included patients on MHD for ≥ 3 months and their caregivers. Patients and/or caregivers with documented dementia were excluded. Through a face-to-face interview, FS was assessed by combining self-reports of 8 instrumental, and 5 basic activities of daily living using the Lawton-Brody and the Katz (LBKQ) scales, and CGB was assessed using the Zarit Caregiver Burden Scale (ZCGBS). We defined functional status impairment (FSI) as a score ≥ 1 on the LBKQ scale, and a high CGB as a ZCGBS score ≥ 41. Data were analysed using the IBM-SPSS version 26.0

**Results:**

A total of 115 patients and 51 caregivers (CGs) were enrolled. The mean age of the patients was 46.9 ± 15.0 years, and 54.8% (*n* = 63) were males, whereas the mean age of the CGs was 38.30 ± 13.10 years with 72.5% (*n* = 39) being females. A total of 90 (78.3%) patients had functional status impairment (FSI), while 78.4% (*n* = 40) of caregivers experienced a burden (41.2% classified as moderate, and 37.2% as high). Anaemia (aOR = 9.2, CI = 3.9–29.4, *p* < 0.001), and a high daily pill burden (aOR = 4.4, CI = 1.1–18.5, p = 0.043) were independently associated with FSI, while age of caregiver ≥ 45 years (aOR 9.9, CI = 1.7–56.8, *P* = 0.01) was independently associated with a high CGB. There was a strong positive correlation between FS and CGB.

**Conclusion:**

There is a high prevalence of functional status impairment in patients on maintenance haemodialysis in Cameroon, resulting in high a physical and psychological burden on their caregivers.

**Supplementary Information:**

The online version contains supplementary material available at 10.1186/s12882-022-02977-3.

## Introduction

The management of End-stage kidney Disease (ESKD) by haemodialysis (HD) significantly increases patient survival [1, 2]. However, the frequent comorbidities, complications of ESKD, complications of HD treatment, and the socioeconomic burden on HD patients may result in poor quality of life, functional status impairment (FSI), and high caregiver burden (CGB) [3, 4].

Functional Status impairment is the inability to perform daily activities required to meet basic needs, fulfill usual roles, and maintain health and well-being [5, 6]. It is highly prevalent in the dialysis population, especially in the elderly [7], with a reported prevalence range of about 32% to 95% [7–11]. The caregiver aids in the patient’s life and most of the medical activities in-between dialysis sessions [12–14]. His or her workload and responsibilities increase when the patient becomes sicker, or has complications. This can result in an increased physical, emotional, and financial burden and stress- caregiver burden [15]. Previous studies have found a high caregiver burden’s prevalence range of 23–78.0% [7, 15–17].

Despite increased survival among patients on maintenance HD, studies have revealed a high prevalence of FSI and CGB [5, 7, 8, 17]. However, these studies involved middle and high-income countries which have an elderly population and optimal dialysis care, in contrast to Cameroon and Sub-Saharan Africa with a younger population, suboptimal dialysis, and high patient out-of-pocket expenditure [18, 19]. Therefore, this study aimed to evaluate the functional status of patients on Maintenance HD (MHD) and their CGB in a low-resource setting. We also explored the sociodemographic and clinical factors associated with FSI and high CGB.

## Methods

### Study setting

This was a hospital-based cross-sectional study conducted in the haemodialysis units of the Bamenda Regional Hospital (BaRH), and the Yaounde General Hospital (YGH) over a period of 3 months (from February 20 to April 28, 2020). These centres all use the Fresenius® 4008S dialysis technology (Fresenius Medical Care, Hamburg, Germany) with synthetic polysulfone dialyzer and bicarbonate dialysate. Each patient benefits from two sessions of four hours each week. Dialysis sessions have been subsidized by the government such that each patient pays a fee of about 10 USD per session. However, this excludes the fee for vascular access, laboratory tests, medications, feeding, transportation, hospitalisation, and vaccination, which are borne by patients and families [19].

During each session, the caregivers wait in the waiting room close by. However, most patients come for their sessions unaccompanied by caregivers; unless they are acutely ill or severely dependent. The caregivers accompany dependent patients to and from the dialysis unit, getting medication from the pharmacy, getting blood for transfusion if needed, running errands to and from the laboratory, and getting food for the patient. They also wait at the unit until the session is over so as to take the patient home or to the ward.

### Study participants and data collection

Consenting patients who had been on MHD for ≥ 3 months and their consenting caregivers (if available) were consecutively enrolled over the study duration. Patients and/or caregivers with documented dementia were excluded.

For each consenting participant, we conducted interviews and reviewed their medical and dialysis records to collect data relevant to our study. Sociodemographic data included: age, sex, religion, level of formal education, residence, and source of funding. Clinical data included: comorbidities, characteristics of ESKD and dialysis, and medication use.

We administered the Katz and Lawton-Brody scales to assess the self-reported dependence of patients in performing 5 activities of daily living (ADL) and 8 instrumental activities of daily living (IADL), respectively. A cumulative score of 0 out of a possible 13 points indicated not care-dependent, 1–5 mild/moderate dependence, and > 5 severe care dependence [7].

Caregiver burden was evaluated using the Zarit Caregiver Burden Scale (ZCBS) [20]. This is a 22-item tool used for the assessment of self-perceived burden in caregivers of sick or aging populations. It has a 5-item response that ranges from “never” to “nearly always”, a score of 0–20 points translating little or no burden, 21–40 meaning a mild to a moderate burden, 41–60 for a moderate to a severe burden, and more than 61 for severe burden.

### Statistical analysis

Statistical analysis was done using the International Business Machines Statistical Package for Social Sciences (IBM-SPSS) version 26.0. Categorical variables were summarised using counts and percentages, presented in tables, and compared using the chi-squared and Fischer’s exact test where appropriate. Continuous variables were summarised using means (with standard deviations), and medians (with interquartile ranges) where necessary, and compared using the student’s t-test. Independent variables associated with functional status impairment and high caregiver burden were analysed by logistic regression, the correlation between functional status and caregiver burden was measured using Spearman's correlation test, and a *p*-value < 0.05 was considered statistically significant. Binary regression was used to identify the predictors of functional status impairment and high caregiver burden in the study population. For multiple logistic regression, we used stepwise regression, and only variables with *p*-values < 0.20 were selected for multiple logistic regression analysis.

The study was conducted in accordance with the Declaration of Helsinki and all participants/legal guardians of minors provided written informed consent before enrolment. The Institutional Review Board (IRB) of the Faculty of Health Sciences of the University of Bamenda approved the study with reference number 2020/0052H7UBa/IRB. Administrative authorizations were obtained from the directors of the Bamenda Regional, and the Yaounde General Hospitals.

### Definition of operational terms

Maintenance haemodialysis was defined as regular haemodialysis treatment for at least 3 months for the management of ESKD. Functional status impairment was defined as an overall functional status assessment scale score ≥ 1. Basic activities of daily living included feeding, dressing, bathing, using the toilet, and transferring from bed to chair. Instrumental activities of daily living were telephone use, getting to places beyond walking distance, shopping, food preparation, light housework, laundry, taking medications, and managing money. Caregivers were designated by the patients. Caregiver burden was defined as a Zarit caregiver burden scale score ≥ 21, and high caregiver burden as a Zarit caregiver burden scale score ≥ 41. Comorbidities were based on documented history or ongoing treatment. Aetiologies of ESKD were based on documented history. Anaemia was defined as a haemoglobin level ˂ 10 g/dl.

## Results

Of the 116 eligible patients in both health facilities, 1 patient was excluded. None caregivers contacted were excluded.

A total of 115 patients on maintenance haemodialysis and 51 caregivers of 51 of these patients were consecutively enrolled in the study.

The mean age ± SD of patients was 46.9 ± 15.0 years, and 63 (54.8%) were males. There were 114 (99.1%) participants who resided within their town of treatment, and 91(79.1%) participants had at least a secondary school education.

Hypertension (*n* = 97, 84.3%), and anaemia (*n* = 86, 74.8%) were the most frequent comorbidities, while hypertension (*n* = 42, 36.5%) and Chronic glomerulonephritis (*n* = 19, 16.5%) were the main known aetiologies of ESKD see Table 1.

**Table 1:** Frequency of comorbidities, and aetiologies of ESKD (*N* = 115)

Comorbidity n(%)	Frequency (%)
Hypertension	97 (84.3)
Anaemia	86 (74.8)
Overweight/obesity	27 (23.5)
Diabetes mellitus	21 (18.3)
Underweight	15 (13.0)
Congestive Heart failure	13 (11.3)
HIV	10 (8.7)
Hepatitis C	7 (6.1)
Hepatitis B	6 (5.2)
Stroke	5 (4.3)
Disability	4 (3.5)
**Aetiology of ESKD. n(%)**	
Hypertension	42 (36.5)
CGN	19 (16.5)
Unknown	18 (15.7)
CIN	10 (8.7)
Diabetes mellitus	9 (7.8)
Others	18 (15.6)

*ESKD* End-stage kidney disease, *CGN* Chronic glomerulonephritis, *CIN* Chronic Interstitial Nephritis, Others: Ischemic Nephropathy = 6, HIV = 4, Obstructive Nephropathy = 1 Autosomal Dominant Polycystic Kidney Disease = 7

The median (25^th^-75^th^ percentile) dialysis vintage was 34 (9–62) months. There were 93 (80.9%) participants who dialysed through an arteriovenous fistula, and 113 (98.3%) participants dialysed twice weekly see Table 2.

**Table 2:** Characteristics of Haemodialysis (*N *=115)

Variable	Frequency (%)
**Median Duration on HD (IQR) in months**	34 (9–62)
**HD Vintage in months**	
< 12 months,	32 (27.8)
12–60 months	53 (46.1)
> 60 months	30 (26.1)
**Type of vascular access**	
Arteriovenous fistula	93 (80.9)
Central venous catheter	22 (19.1)
**Number of weekly sessions**	
2	113 (98.3)
1	2 (1.7)
**Frequent Intradialytic complications**	
Muscle cramps	81 (70.4)
Headache	28 (24.3)
Nausea and vomiting	23 (20.0)
Intradialytic hypotension	14 (12.2)

*HD* Haemodialysis, *IQR* Interquartile range

A total of 80 (69.6%) participants were on antihypertensives; vitamin D analogues and erythropoietin were being used by 25.3% (*n* = 29) and 18.3% (*n* = 21) of participants respectively. The median (25^th^ -75^th^) number of pills taken daily by participants was 2 [1–6], and 38.3% (*n* = 44 of participants) took at least four pills daily see Table 3.

**Table 3:** Medication use among patients (*N* = 115)

Variable	Frequency (%)
**Antihypertensives**	80 (69.6)
CCB	65 (56.5)
Beta-blockers	35 (30.4)
ACEI	32 (27.8)
ARB	17 (14.8)
Alpha-2-agonists	10 (8.7)
**Other Medications**	
Vitamin D analogues	29 (25.2)
Erythropoietin	21 (18.3)
**Daily pill burden**	
0	21 (18.2)
1–3	50 (43.5)
≥ 4	44 (38.3)

*CCB* Calcium Channel Blockers, *ACEI* Angiotensin-Converting Enzyme Inhibitors, *ARB* Angiotensin Receptor Blockers

Concerning caregivers, their mean age ± SD was 38.9 ± 13.1 years, and females accounted for 72.5% (*n* = 39). Spouses and children accounted for about two-thirds of caregivers (*n* = 35, 68.6%), and 90% (*n* = 18) of spouses were wives. There were 35 (68.6%) caregivers who parented dependent children.

The median (25^th^-75^th^ percentile) functional status score was 3 (1-5) with a range of 0–12, and no significant difference between the two sites [BaRH:3.0 (1-5) vs YGH:3.0 (0–4), *p* = 0.745].

The prevalence of functional status impairment was 78.3% (*n* = 90), with 24.4% (*n* = 22) classified as severe. All participants with FSI experienced impairment in the domain of instrumental activities of daily living, while 22 (24.4%) of them experienced impairment in basic activities of daily living.

**Fig. 1:**
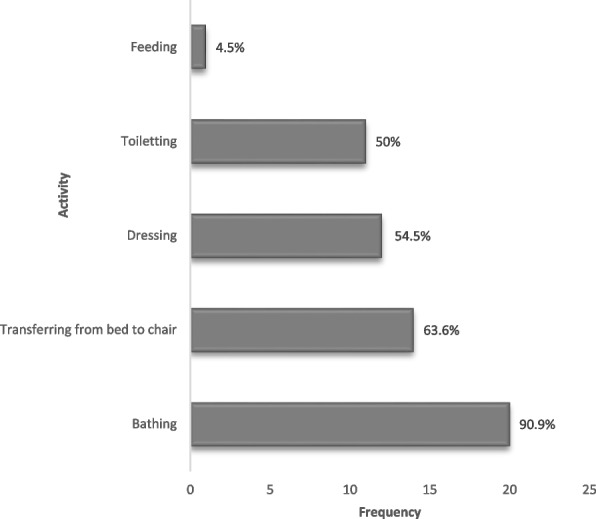
Distribution of domains of impairment in basic activities of daily living (*n* = 22)

**Fig. 2:**
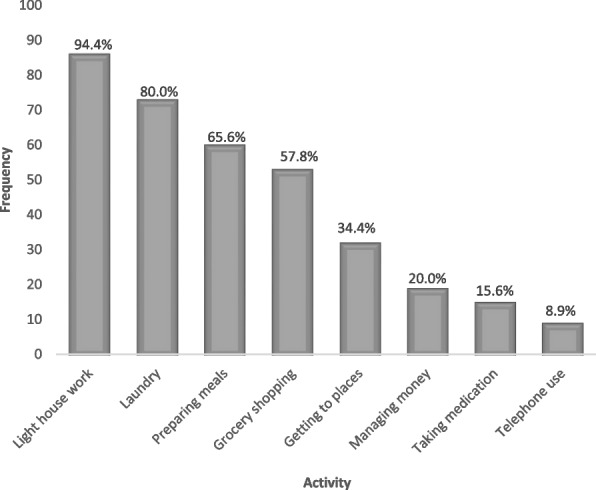
Distribution of domains of impairment in instrumental activities of daily living (*n* = 90)

Bathing (*n* = 20, 90.9%), transferring from bed to chair (*n* = 14, 63.6%), and dressing (*n* = 12, 54.5%) were the most impaired basic activities of daily living (Fig. 1), whereas light housework (*n* = 85, 94.4%), laundry (*n* = 72, 80.0%), preparing meals, (*n* = 59, 65.6%), and grocery shopping (*n* = 52, 57.8%) were the main instrumental activities of daily living impaired (Fig. 2).

Overall, 40 (78.4%) caregivers experienced a burden; 22 caregivers (41.2%) experienced a mild to moderate burden, and 19 (37.3%) experienced a high burden.

Lack of funds for patient care (median 3, IQR 2–4), anxiety about the future of their patient (median 3, IQR 2–3), and the stress of caring for patients and meeting other needs (median 2, IQR 2–3) were the main items involved. However, caregivers still desired to do more for their patients (median 2, IQR 2–3).

### Factors associated with functional status impairment and high caregiver burden

On univariate analysis, age > 30 years (*p* = 0.002, 0R = 5.8), the presence of anaemia (*p* = 0.001, OR = 10.5), and high daily pill intake (*p* = 0.005, OR = 6.1) significantly increased the odds of having FSI. After adjustment, anaemia (*p* < 0.001, aOR = 9.2), and a high daily pill burden (*p* = 0.043, aOR = 4.4) were independently associated with functional status impairment. See Table 4.

**Table 4:** Factors associated with FSI (Multivariate Analysis)

Factor	Adjusted OR (95% CI)	*p*-value
Anaemia	9.2 (3.0–29.4)	< 0.001
High daily pill intake(≥ 4)	4.4 (1.1–18.5)	0.043
Age ≥ 30 years	1.3(0.3–6.3)	0.066
Obesity/ overweight	2.7 (0.5–14.7)	0.254
Congestive heart failure	3.5 (0.2–58.3)	0.380
Diabetes	2.3 (0.2–25.5)	0.489
< Secondary Education	1.3 (0.3–6.3)	0.742
Hypertension	1.2 (0.3–4.9)	0.818
Sex(male)	1.1 (0.3–3.8)	0.833

Age ≥ 45 years (*p* = 0.01, aOR = 9.9) was independently associated with high caregiver burden) see Table 5.

**Table 5:** Factors associated with High Caregiver burden (Multivariate analysis)

Variable	Adjusted OR (95% CI)	*p*-value
Age ≥ 45 years	9.9(1.7–56.8)	**0.01**
Female sex	3.0(0.5–16.5)	0.217
Presence of dependent children	1.4 (0.3–7.6)	0.692
Spousal Caregivers	1.1(0.2–5.2)	0.904
< Secondary Education	1.1(0.1–8.1)	0.954

There was a significant strong positive correlation between functional status score, and caregiver burden score (*r* = 0.6, *p* < 0.001) indicating that caregiver burden significantly increased with increasing functional status impairment.

## Discussion

In this study, we sought to determine the sociodemographic and clinical factors associated with functional status impairment and the high caregiver burden of patients on maintenance haemodialysis in a low-resource setting with younger patients who receive sub-optimal dialysis care and pay out-of-pocket for dialysis-related costs. We found a high 78.3% prevalence of functional status impairment and a 78.4% prevalence of caregiver burden. Anaemia and high daily pill burden were independently associated with FSI, whereas only age ≥ 45 years was independently associated with a high caregiver burden. We found caregiver burden to be strongly correlated with functional status impairment.

The prevalence of functional status impairment in the dialysis population varies from 32–95% [8–10, 7, 11]. We found a high prevalence of 78.3%. This agrees with previous studies conducted in the Netherlands (79%) and the International Dialysis Outcome Practice Pattern Study (64%) [7, 10]. Factors that could account for the high prevalence in our setting can include multiple comorbidities, suboptimal dialysis, anaemia, depression, economic and psychosocial issues, or the physiologic impact of persistent uraemia [3]. Also, post-dialysis fatigue and rapid volume shifts may have an impact on overall health and functionality, and the observation that both caregivers and healthcare workers facilitate patients taking on learned helplessness may perpetuate the decline in physical health and self-care ability [10]. This high prevalence of functional impairment may contribute to the poor quality of life and high morbidity seen in dialysis patients. Moreover, greater functional dependence is strongly associated with dialysis withdrawal [10] and increased death rates [3, 21]. Besides, functional dependence, independent of its cause is associated with high healthcare costs and increased risk of hospitalisation [22]. This suggests the possible need for care that includes close attention to modifiable symptoms, such as pain, fatigue, and nausea, in an attempt to improve functional status, and highlights the discussion on the value of care that prioritizes symptom management over laboratory-target–driven dialysis care [10]. Our prevalence was lower than the 95.1% found by Cook et al. in patients aged ≥ 65 years in Canada [11]. This may be explained by our younger population. The elderly population tends to have more comorbidities, and elderly age is associated with FSI [8, 21]. Our prevalence was however more than the 32% found by Akash et al. in Jordan. In contrast to their study, we included patients on haemodialysis for at least 3 months in contrast to theirs of 12 months. Moreover, their patients had more weekly dialysis doses. The longer duration of Haemodialysis and more frequent weekly sessions are associated with better functional status [23].

The caregivers of dialysis patients face various challenges and complex problems due to emotional, economic, cognitive, and social limitations from ESKD [24]. We found that 78.4% of caregivers experienced a burden in caregiving, with 37.2% as above moderate. This is similar to the rates found in other countries [15–17]. Our prevalence was however higher than the 23% among caregivers of patients in the Netherlands at the initiation of dialysis, but it increased to 38% after 6 months [7]. It is now well recognized that care for patients with chronic fatal illnesses by family members has two-opposite impacts on the physical and mental health of caregivers. One is positive because the obligation and responsibility to take care of a loved one may cause caregivers to experience positive aspects of care such as satisfaction, rewards, and enjoyment and give the caregiver all the inspiration to give his/her best [4, 15]. However, long-term caregiving also has a significantly negative influence. The physical, emotional, economical, and timing strains may overwhelm the caregivers [15]. Our study also revealed that the total burden was largely driven by the lack of funds by caregivers, the anxious feeling about what the future holds for their patients, and the stress between caring for patients and meeting other needs. However, they still desired to do more for their patients. This may be a pointer to a deep concern about the health of their patient. Factors that are associated with functional status impairment in patients on maintenance haemodialysis include older age, female sex, multiple comorbidities, the presence of diabetes, obesity, anaemia, etc. [3, 8–10]. In this study, anaemia and a high daily pill burden were independently associated with impaired functional status. Saha et al. found a positive correlation between higher haemoglobin levels and better quality of life [25], while correction of anaemia, even sub-optimally by the use of EPO improved physical function and energy [26]. The association with a high daily pill burden may be explained by the fact that patients with an impaired functional status usually have multiple comorbidities and may therefore have multiple drug prescriptions. Moreover, the interactions and side effects of these drugs may contribute to their functional status impairment. Participants with functional status impairment were significantly older than those without.

Some factors have been associated with caregiver burdens such as older age, female sex, low socioeconomic status, low level of education, being a spouse to the patient, parent, and adult-children caregivers, and more caregiving hours and tasks [4, 15, 17]. In this study, we found only age ≥ 45 years to be significantly associated with high caregiver burden. This may be explained by the fact that middle-aged caregivers are also breadwinners, and have other responsibilities, interruptions at work, and reduced productivity due to caregiving [4].

Caregivers of patients who need more assistance in their daily life experience more burden [4, 17]. In this study, we found caregiver burden to significantly increase with functional status impairment. The provision of long-term daily care to patients undergoing haemodialysis leads to a physical and psychological burden, which increases with the level of dependence of the patients. Caregivers are less burdened if patients are independent in their everyday activities and have fewer complaints about dialysis treatment [4].

### Strengths and limitations

Our study has some limitations. Firstly, the sample size appears small hence the results of our study may not be generalizable to the whole population of patients on MHD and their caregivers in Cameroon. However, to the best of our knowledge, this is the first study in our country, and sub-Saharan Africa on functional status impairment and caregiver burden of patients on maintenance haemodialysis, and may serve as a building block for further studies.

## Conclusion

In conclusion, there is a high 78.4% prevalence of functional status impairment in patients on maintenance haemodialysis and a high 78.3% prevalence of caregiver burden in Cameroon. Anaemia and a high daily pill burden are associated with functional status impairment, while a caregiver age ≥ 45 years is associated with a high caregiver burden. In addition, caregiver burden strongly correlates with functional status impairment.

## Supplementary Information

**Additional file 1:** **Table S1. **Factors associated with Functional status impairment on Univariate Analysis (*N*=115). **Table S2. **Socio-demographic factors associated with high caregiver burden on Univariate Analysis (*N*=51).

## Data Availability

The datasets used and/or analysed during the current study are available from the corresponding author upon reasonable request. The datasets generated and analysed during the current study are not publicly available because the hospitals do not permit the researchers to share the datasets publicly.

## Abbreviations

ADL: Activities of daily living
aOR: Adjusted Odds Ratio
BaRH: Bamenda Regional Hospital
CG: Caregivers
CGB: Caregiver burden
CI: Confidence interval
ESKD: End-stage kidney Disease
FS: Functional status
FSI: Functional status impairment
HCGB: High caregiver burden
HD: Haemodialysis
IADL: Instrumental activities of daily living
IQR: Interquartile range
IRB: Institutional Review Board
LBKQ: Lawton-Brody and the Katz scale
MHD: Maintenance haemodialysis
YGH: Yaounde General Hospital
ZCGBS: Zarit Caregiver Burden Scale
