# Supplementary material for: Functional status and caregiver burden of patients on maintenance haemodialysis in Cameroon: a two-centre cross-sectional study
Source: BMC Nephrol. 2022 Oct 22;23:341. doi: 10.1186/s12882-022-02977-3 (PMC9587608; doi:10.1186/s12882-022-02977-3)
Supplement: Supplementary file 1 — Additional file 1: Table S1. Factors associated with Functional status impairment on Univariate Analysis (N=115). Table S2. Socio-demographic factors associated with high caregiver burden on Univariate Analysis (N=51). [file 12882_2022_2977_MOESM1_ESM.doc]

| **Table S1: Factors associated with Functional status impairment on Univariate Analysis (N=115)** | | | | |
| --- | --- | --- | --- | --- |
| **Variable** | Presence of FSI | | OR(95% CI) | *p*-value |
|  | Yes  n=90 | No  n=25 |  |  |
| **Age ranges(years):** |  |  |  |  |
| **≥30** | 82 (91.1) | 16(64.0) | 5.8(1.9-17.2) | 0.002 |
| **<30** | 8(8.9) | 9(36.0) | 1 |  |
| **Sex** |  |  |  |  |
| **Male,** | 52 (57.8) | 11 (44.0) | 1.7 (0.7-4.3) | 0.224 |
| **Female** | 38 (42.2) | 14 (56.0) | 1 |  |
| **Level of formal education** | | | | |
| **<Secondary** | 21(23.3) | 3(12.0) | 2.2 (0.6-8.2) | 0.227 |
| **≥Secondary, n (%)** | 69(76.7) | 22 (88.0) | 1 |  |
| **Source of funding n(%)** | | | | |
| **Family** | 67 (74.4) | 16 (64.0) | 1.6 (0.6-4.2) | 0.305 |
| **Others** | 23 (25.6) | 9 (36.0) | 1 |  |
| **Hypertension** |  |  |  |  |
| **Yes** | 78(86.7) | 19(76.0) | 2.1(0.7-6.2) | 0.200 |
| **No** | 12(13.3) | 6(24.0) | 1 |  |
| **Diabetes mellitus** |  |  |  |  |
| Yes | 20(22.2) | 1(4.0) | 6.9(0.9-53.9) | 0.067 |
| No | 70(77.8) | 24(96.0) | 1 |  |
| **Anaemia** |  |  |  |  |
| **Yes** | 77(85.6) | 9(36.0) | 10.5(3.9-28.8) | <0.001 |
| **No** | 13(14.4) | 16(64.0) | 1 |  |
| **Obesity/overweight** |  |  |  |  |
| **Yes** | 24(26.7) | 3(12.0) | 2.7(0.7-9.7) | 0.137 |
| **No** | 66(73.3) | 22(88.0) | 1 |  |
| **Underweight** |  |  |  |  |
| **Yes** | 13(14.4) | 2(8.0) | 1.9(0.4-9.2) | 0.518 |
| **No** | 77(85.6) | 23(92.0) | 1 |  |
| **Congestive Heart failure** | | | | |
| **Yes** | 12(13.3) | 1(4.0) | 3.7(0.5-29.9) | 0.220 |
| **No** | 78(86.7) | 24(96.0) | 1 |  |
| **HIV** |  |  |  |  |
| **Yes** | 8(8.8) | 2(8.0) | 1.1(0.2-5.7) | 1.00 |
| **No** | 82(91.2) | 23(92.0) | 1 |  |
| **Daily pill intake** |  |  |  |  |
| **≥4** | 41(45.6) | 3(12.0) | 6.1(1.7-22.0) | 0.005 |
| **<4** | 49(54.4) | 22(88.0) | 1 |  |
| **OR**: Odds Ratio; **CI**: Confidence Interval | | | | |

| **Table S2: Socio-demographic factors associated with high caregiver burden on Univariate Analysis (N=51)** | | | | | |
| --- | --- | --- | --- | --- | --- |
| **Variables** | | **Presence of high burden** | | **OR(95% CI)** | ***p*-value** |
|  |  | Yes  n=19 | No  n=32 |  |  |
| **Age ranges in years) n(%)** | | | | | |
| **≥45** | 11(57.9) | | 4(12.5) | 9.6(2.4-38.6) | **0.001** |
| **<45** | 8(42.1) | | 28(87.5) | 1 |  |
| **Sex n(%)** | | | | | |
| **Female** | 16(84.2) | | 21(65.6) | 2.8(0.7-11.7) | 0.160 |
| **Male** | 3(!5.8) | | 11(34.4) | 1 |  |
| **Level of formal education n (%)** | | | | | |
| **<secondary** | 7(36.8) | | 4(12.5) | 4.1(1.01-16.6) | **0.049** |
| **≥secondary** | 12(63.2) | | 28(87.5) | 1 |  |
| **Caregiver-patient relationship n(%)** | | | | | |
| **Spouse** | 10(52.6) | | 10(31.3) | 2.4(0.8-7.9) | 0.13 |
| **Others** | 9(47.4) | | 22(68.8) | 1 |  |
| **Parent of Dependent Children n(%)** | | | | | |
| **Yes** | 15(78.9) | | 20(62.5) | 2.3(0.6-8.4) | 0.23 |
| **No** | 4(21.05) | | 12(37.5) | 1 |  |
| **OR**: Odds Ratio; **CI**: Confidence Interval | | | | | |
